# Case report of pyloric obstruction caused by giant gallbladder compression with literature review

**DOI:** 10.3389/fsurg.2026.1727900

**Published:** 2026-01-26

**Authors:** Wenhui Xu, Panpan Liu, Qinyu Ni, Xuedong Cao, Kun Liu, Shigui Xue, Yueqiu Gao, Xiaojun Zhu

**Affiliations:** 1 Department of Hepatology, Shuguang Hospital Affiliated to Shanghai University of Traditional Chinese Medicine, Shanghai, China; 2 Department of Hepatobiliary and Pancreatic Surgery, Shuguang Hospital Affiliated to Shanghai University of Traditional Chinese Medicine, Shanghai, China; 3 Department of Pathology, Shuguang Hospital Affiliated to Shanghai University of Traditional Chinese Medicine, Shanghai, China; 4 Department of Endoscopy, Shuguang Hospital Affiliated to Shanghai University of Traditional Chinese Medicine, Shanghai, China

**Keywords:** case report, cholelithiasis, chronic cholecystitis, giant gallbladder, laparoscopic cholecystectomy, pyloric obstruction

## Abstract

Giant gallbladder is a rare clinical condition characterized by abnormal gallbladder enlargement, typically defined as a longitudinal diameter exceeding 14 cm or a volume surpassing 1.5 L. The most common pathological etiologies are cholelithiasis and chronic cholecystitis, followed by neoplastic factors, while congenital developmental anomalies and other causes are relatively uncommon. Herein, we report a case of giant gallbladder-induced pyloric obstruction in a patient who presented with nausea and vomiting for two weeks—symptoms that recurred after initial resolution of diabetic ketoacidosis. Abdominal computed tomography (CT) revealed a giant gallbladder with cholelithiasis, and gastroscopy demonstrated an extrinsic compressive bulge in the gastric antrum plus narrowing at the second and third part of duodenum (D2 & D3).The patient was diagnosed with cholelithiasis and chronic cholecystitis leading to giant gallbladder, which caused pyloric obstruction via compression. Management involved initial ultrasound-guided percutaneous gallbladder drainage, followed by laparoscopic cholecystectomy (LC) one week later, and the postoperative course was uneventful. This case highlights that giant gallbladder may present with atypical gastrointestinal symptoms (e.g., isolated nausea and vomiting) and is prone to misdiagnosis, especially in middle-aged and elderly females with comorbidities. Confirmation of the compressive mechanism requires integration of imaging and endoscopic findings; for patients with giant gallbladder complicated by severe adhesions or underlying comorbidities, a two-stage surgical approach (initial decompressive drainage followed by laparoscopic excision) is a safe and effective option.

## Introduction

The normal gallbladder is a piriform-shaped sac, approximately 7–10 cm in length, with an average capacity of 30–50 mL. Under obstructive conditions, the gallbladder may distend, accommodating up to 350 mL of bile (1). Giant gallbladder is a clinically rare entity, and currently, there is no unified classification criteria. One study proposed diagnostic criteria of a longitudinal diameter exceeding 14 cm or a volume surpassing 1.5 L (2). Another study defined it based on gallbladder weight exceeding that of the liver (approximately 1.5 kg) (3). Giant gallbladder is an uncommon clinical condition, most frequently caused by cholelithiasis, acute or chronic cholecystitis, and typically presents with classic symptoms such as right upper quadrant pain. Reports describing cases presenting primarily with isolated nausea and vomiting are scarce. Herein, we report a case of pyloric obstruction caused by compression from a giant gallbladder and describe the clinical diagnosis and management process for this patient. This case report adheres to the CARE Guidelines (CARE: Case Report Guidelines).

## Case presentation

**Figure 1:**
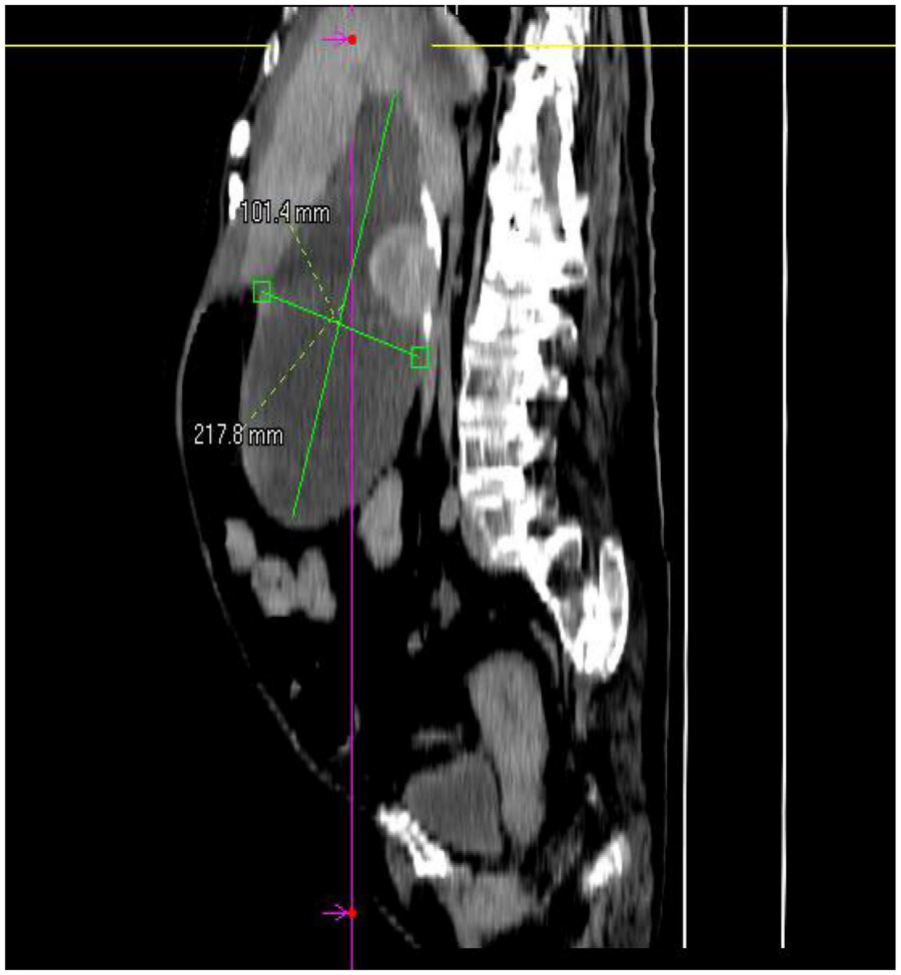
Whole-abdominal CT shows: the gallbladder has a long diameter of 217.8 mm and a transverse diameter of 101.4 mm.

**Figure 2:**
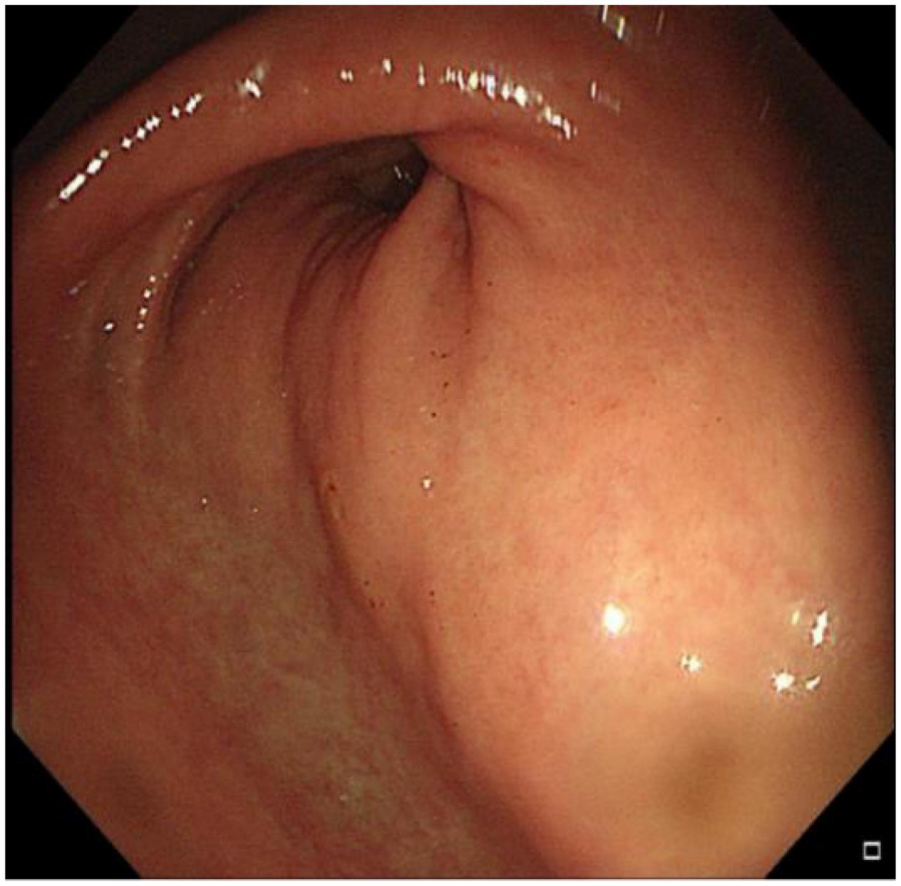
Gastroscopy reveals a large extrinsic compression bulge in the gastric antrum.

**Figure 3:**
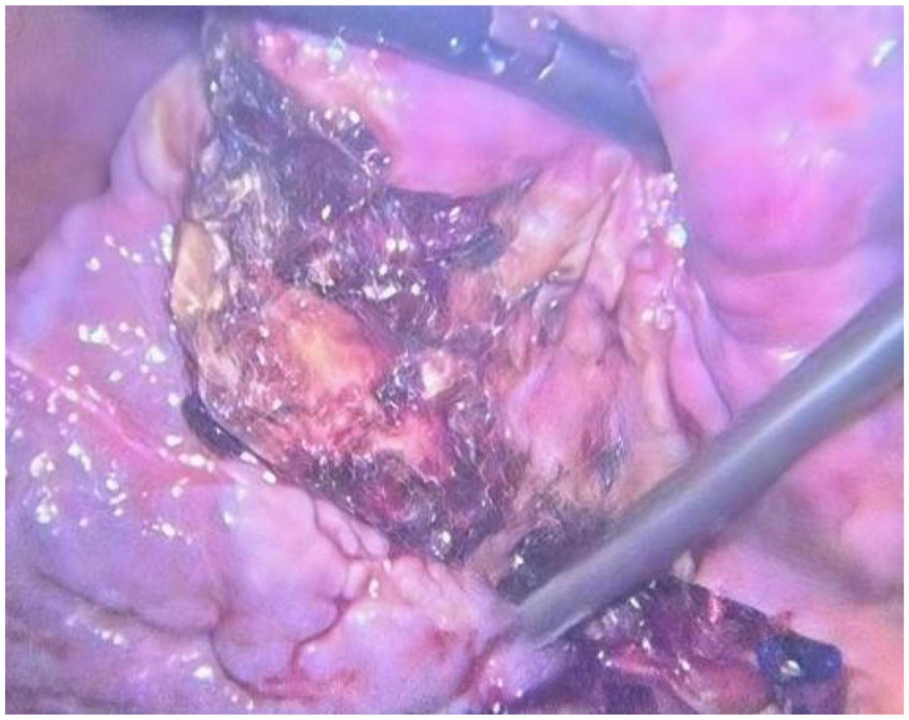
Laparoscopy shows liver cirrhosis and the gallbladder fossa after cholecystectomy.

**Figure 4:**
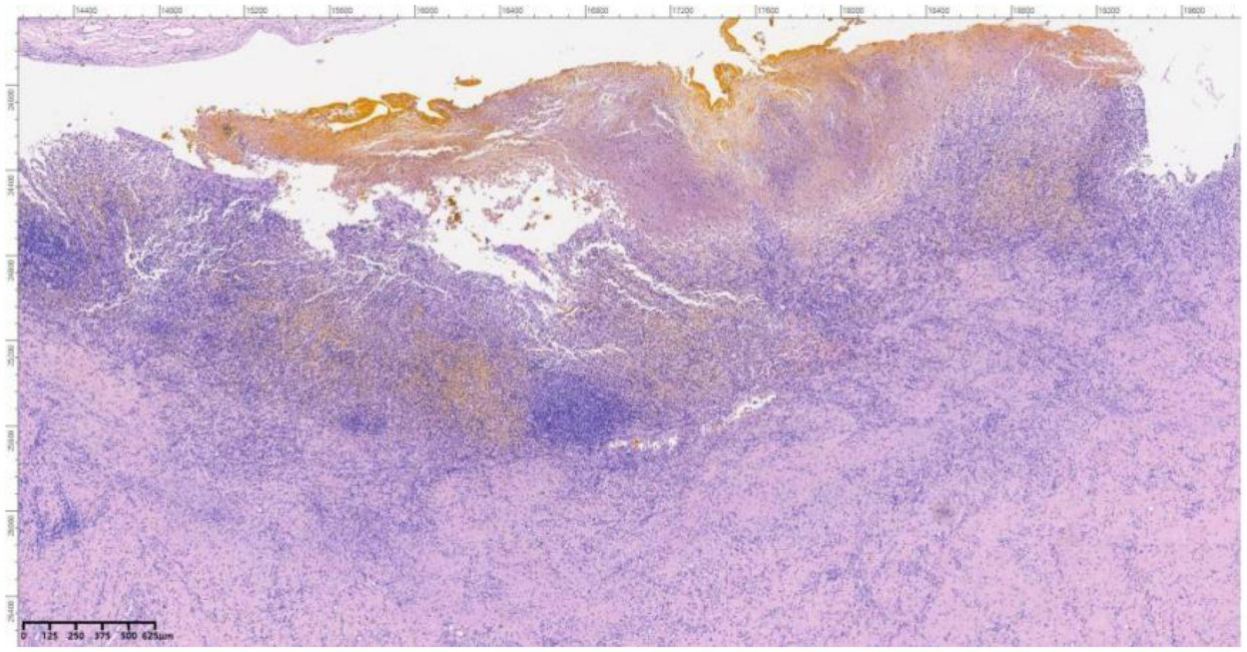
Objective lens magnification (3.30×): visible mucosal necrosis and desquamation, with fibrous thickening of the gallbladder wall accompanied by a large number of chronic inflammatory cells. (19)

**Figure 5:**
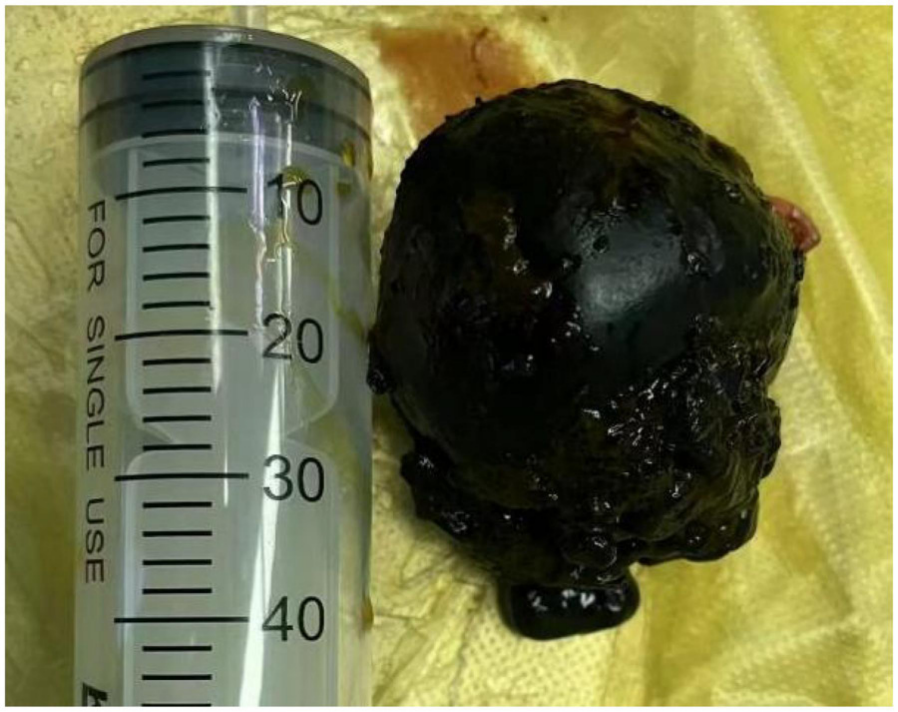
Gallbladder stones after surgical resection.

A 69-year-old female presented to the Department of Hepatology in our hospital with “intermittent nausea and vomiting for 2 weeks”. She had a 3-year history of cryptogenic cirrhosis without overt portal hypertension and a 5-year history of diabetes mellitus.Prior to this visit, she had undergone open reduction and screw internal fixation in our hospital for a femoral fracture. At presentation, she had no fever; laboratory tests showed elevated blood glucose and ketone bodies. A preliminary consideration was that the nausea and vomiting might be caused by diabetic ketosis, so a consultation with the Department of Endocrinology was requested, and symptomatic treatment was administered, which relieved her vomiting. However, the symptoms recurred 2 days later, at which time blood glucose and ketone bodies had returned to normal. Physical examination revealed abdominal distension, without abdominal bloating, tenderness, or rebound tenderness. Abdominal CT revealed a markedly enlarged gallbladder (approximately 21 cm in longitudinal diameter) containing multiple stones (Figure 1), along with features suggestive of gastric retention and dilatation. Gastric lavage via a nasogastric tube revealed the presence of gastric contents. Further gastroscopy revealed a huge extrinsic bulge compressing the gastric antrum, accompanied by mild narrowing of the duodenum at the D2/D3 junction due to external compression (as shown in Figure 2). Integrating the abdominal CT findings of a giant gallbladder with gastric distension and the gastroscopic evidence of extrinsic compression at the gastric antrum, a multidisciplinary consultation involving gastrointestinal surgery, gastroenterology, and hepatobiliary-pancreatic surgery concluded that the patient's nausea and vomiting were most likely attributable to pyloric obstruction caused by mechanical compression from the giant gallbladder on the gastric antrum and duodenum. Accordingly, considering her test results (Table 1A) and physical condition, a two-stage treatment plan was formulated: initial ultrasound-guided percutaneous transhepatic gallbladder drainage to decompress the gallbladder and relieve its compressive obstruction on the gastrointestinal tract, followed by elective laparoscopic cholecystectomy once the patient's condition stabilized. After approximately 500 mL of bile was drained via gallbladder puncture, the patient's clinical symptoms of nausea and vomiting were immediately relieved. Repeated gastric lavage via the nasogastric tube showed no gastric contents could be aspirated, so we judged that the pyloric obstruction caused by compression of the duodenum by the giant gallbladder had been relieved. The patient was discharged for 1 week of rest; after her physical strength and nutritional status recovered, she was re-admitted to the Department of Hepatobiliary and Pancreatic Surgery for laparoscopic cholecystectomy. Following admission to the Department of Hepatobiliary and Pancreatic Surgery for preoperative workup, the patient was found to have no relevant surgical contraindications. She was asymptomatic, with all laboratory results within acceptable limits (Table 1B) and a Child-Pugh class A score. Based on this evaluation, laparoscopic cholecystectomy was approved. As preoperative Magnetic Resonance Cholangiopancreatography (MRCP) had already ruled out biliary stones or obstruction, intraoperative cholangiography was omitted.Intraoperative exploration revealed marked liver cirrhosis, a significantly enlarged gallbladder, adhesions with the surrounding greater omentum and adhesions of the gallbladder to the gastric antrum and the upper segment of the duodenum. The chief surgeon successfully performed laparoscopic cholecystectomy, and the postoperative view revealed the gallbladder bed following resection (Figure 3). Postoperative pathology showed, at an objective magnification of 3.30×, mucosal necrosis and exfoliation, fibrous thickening of the gallbladder wall with a large number of chronic inflammatory cells (as shown in Figure 4). The gallbladder measured 15 cm × 7 cm × 1.5 cm, with disappearance of gallbladder mucosal folds, a wall thickness of 0.2–0.4 cm, and a black round stone with a diameter of 5 cm inside the gallbladder (as shown in Figure 5). The diagnosis was cholelithiasis and chronic cholecystitis. After the surgeon successfully completed the laparoscopic cholecystectomy, she was able to resume diet on the second day and was discharged on the 3rd postoperative day. At the 2-week postoperative follow-up, the patient reported good recovery with no complications.

**Table 1A:** Pre-procedural laboratory tests for percutaneous gallbladder puncture.

Laboratory test	Result	Unit	Reference range
Complete Blood Count			
WBC	4.53	10^9/L	3.97–9.15
Hb	78.0	g/L	115–150
CRP	53.60	mg/L	0.00–8.00
Liver Function Tests			
TBil	29.5	umol/L	0.0–23.0
DBil	10.0	umol/L	0.0–4.0
ALT	8	U/L	9–50
AST	15	U/L	15–40
ALP	96	U/L	45–125
Alb	28.5	g/L	40.0–55.0
Renal Function Tests			
Cr	79	umol/L	57–97
UA	296	umol/L	208–428
Coagulation Profile			
PT	16.0	S	10.4–12.7
INR	1.37		0.85–1.15
Electrolytes			
K	4.27	mmol/L	3.50–5.10
Na	137	mmol/L	136–146
Glu	5.5	mmol/L	4.3–5.9

**Table 1B:** Pre-operative laboratory tests—laparoscopic cholecystectomy.

Laboratory test	Result	Unit	Reference range
Complete blood count			
WBC	4.64	10^9/L	3.97–9.15
Hb	84.0	g/L	115–150
CRP	40.40	mg/L	0.00–8.00
Liver function tests			
TBil	40.3	umol/L	0.0–23.0
DBil	11.1	umol/L	0.0–4.0
ALT	13	U/L	9–50
AST	20	U/L	15–40
ALP	119	U/L	45–125
Alb	38.0	g/L	40.0–55.0
Renal function tests			
Cr	143	umol/L	57–97
UA	595	umol/L	208–428
Coagulation profile			
PT	13.5	S	10.4–12.7
INR	1.14		0.85–1.15
Electrolytes			
K	3.88	mmol/L	3.50–5.10
Na	140	mmol/L	136–146
Glu	4.5	mmol/L	4.3–5.9

## Discussion

This study conducted a search on PubMed using “Giant Gallbladder” as the keyword and defined a giant gallbladder as one with a long diameter exceeding 14 cm. Clinical reports on giant gallbladder published from 2014 to the present were screened, and 13 relevant studies were identified (see Table 2). Among these clinical cases of giant gallbladder, 12 cases were complicated with cholelithiasis, including 6 cases of acute cholecystitis (with 1 case of gallbladder perforation (4) and 1 case of gallbladder empyema (5); 4 cases of chronic cholecystitis (with 1 case of gallbladder hydrops (6) and 1 case of duodenal compression (7). Additionally, 2 cases were diagnosed as neoplastic lesions of the gallbladder. There was 1 case of congenital gallbladder enlargement with no stones found in the gallbladder (8). Statistical analysis showed that among all 13 patients with giant gallbladder, the number of female patients (11 cases, 84.6%) was significantly higher than that of male patients (2 cases, 15.4%), indicating that females are at a higher risk of developing giant gallbladder. The age distribution of patients with giant gallbladder was relatively wide, ranging from 36 to 85 years, with a mean age of 61.23 years, which is generally in the upper middle age range. Based on the available data, it can be cautiously inferred that females and middle-aged and elderly populations may be relatively high-risk groups for giant gallbladder.

**Table 2:** Summary of clinical characteristics in reported cases of giant gallbladder..

Report	Age	Gender	Symptoms	Diagnosis	Physical examination	Gallbladder Size	Stones	Treatent plan	Histopathology
(2)	63	Female	Right lower abdominal pain and swelling	Giant gallbladder stones complicated with acute cholecystitis	A mass palpable in the right iliac fossa	19.5 × 5.4 × 5.6 cm	Multiple stones	Mini-laparoscopic cholecystectomy with extended umbilical incision	Accumulation of hemophagocytic cells in the wall, mixed with foreign body-type multinucleated giant cells
(4)	57	Female	Fever, chills, right upper abdominal pain, radiating pain to the scapula	Giant gallbladder stone with gallbladder perforation and liver abscess	Tenderness and muscle tension in the upper abdominal region	15 × 12 cm	Single stone	Open subtotal cholecystectomy, endoscopic retrograde cholangiopancreatography (ERCP), common bile duct stenting	Xanthogranulomatous cholecystitis
(5)	42	Male	Scleral icterus, abdominal distension	Giant empyema of the gallbladder complicated with cholelithiasis	Not reported	30 × 20 × 30 cm	Multiple stones	Laparoscopy converted to midline open cholecystectomy	Missing
(6)	53	Female	Acute right upper abdominal pain	Hydrops cholecystitis complicated with cholelithiasis	An abdominal mass palpable in the right hypochondriac region, extending to the upper part of the right iliac fossa	22 × 14 × 10 cm	Multiple stones	Open cholecystectomy	Chronic cholecystitis
(7)	82	Male	Anorexia	Giant gallbladder with stones and duodenal compression	A mass palpable in the right hypochondriac region	20 × 7 cm	Multiple stones	Laparoscopic cholecystectomy	Gallbladder wall fibrosis and chronic cholecystitis
(8)	47	Female	Right upper abdominal mass with dull pain	Congenital gallbladder enlargement	A cystic mass palpable in the right upper abdomen, non-tender, extending to the right iliac fossa	27 × 25 × 12 cm	None	Open cholecystectomy	Chronic inflammation of the gallbladder wall
(9)	36	Female	Progressive aggravation of right upper abdominal pain, accompanied by nausea, vomiting and anorexia	Giant gallbladder with stones complicated with acute cholecystitis	Tenderness in the right upper abdomen, gallbladder palpable, Murphy's sign positive	22 × 6 cm	Multiple stones	Open cholecystectomy	Chronic gangrenous cholecystitis
(10)	65	Female	Progressive swelling of the right abdomen	Giant gallbladder tumor	A solid mass palpable in the right abdomen, extending from the right hypochondriac region to the right iliac fossa	23 × 8 cm	Multiple stones	Diagnostic laparoscopy+open radical cholecystectomy+wedge liver resection+regional lymph node dissection	Well-differentiated gallbladder adenocarcinoma [Stage II (T2a N0 M0)]
(11)	71	Female	A sense of pressure from the mass in the right upper abdomen, frequent urination	Giant gallbladder with stones complicated with chronic cholecystitis	Abdomen soft without tenderness, fullness palpable under the right costal arch	30 × 15 cm	Multiple gallstones	Open cholecystectomy	Missing
(12)	77	Female	Progressive swelling of the right abdomen, anorexia, weight loss	Giant gallbladder tumor	A hard solid mass palpable in the right hypochondriac region and right iliac fossa	25 × 7 cm	Multiple gallstones	Midline open radical cholecystectomy, wedge liver resection, local regional lymph node dissection	Biliary intraductal-tubular tumor of the gallbladder [pT2aN0M0]
(13)	85	Female	Anorexia, right lower abdominal pain	Giant gallbladder cyst complicated with acute cholecystitis	Not reported	14.5 × 14.5 × 8.7 cm	Single stone	Laparoscopic cholecystectomy converted to open cholecystectomy after percutaneous transhepatic gallbladder drainage	Inflammation of the gallbladder wall and thickening of the wall of the extracystic cystic lesion
(17)	46	Female	Right lower abdominal pain	Giant gallbladder with stones complicated with chronic cholecystitis	A long tubular mass palpable in the right lower abdomen	20 × 7 cm	Multiple gallstones	Laparoscopic cholecystectomy after gallbladder decompression	Chronic calculous cholecystitis
(18)	72 years old	Female	Biliary colic after eating greasy food	Giant gallbladder stone complicated with acute cholecystitis	Mild tenderness of the gallbladder, palpable, no jaundice	14 × 7 cm	Single stone	Laparoscopic cholecystectomy with extended umbilical incision	Cholesterol stone

The clinical manifestations of patients with giant gallbladder are diverse. Typical symptoms include pain in the right upper abdomen or right hypochondriac region, less common digestive system dysfunctions such as nausea, vomiting, and anorexia (9). A few patients may be accompanied by jaundice due to extrahepatic biliary obstruction (4), as well as special symptoms such as a sense of right abdominal swelling (10), frequent urination (11), emaciation (12), and duodenal compression (7). A typical feature of abdominal palpation in patients with giant gallbladder is a palpable gallbladder or mass in the right upper abdomen, and the mass in some patients can extend to the right iliac fossa (10). However, the onset characteristics of the giant gallbladder patient in this case were distinct: there was no pain in the right abdomen, with only symptoms of nausea and vomiting. Physical examination only revealed abdominal protuberance, without abdominal bloating, abdominal tenderness, or rebound tenderness, and Murphy's sign was negative. These features posed certain difficulties for our clinical diagnosis.

The pathological causes of giant gallbladder (GGB) are complex, involving the interaction of multiple factors. According to previous literature reports, the pathology of giant gallbladder is centered on cholelithiasis and acute or chronic inflammation of the gallbladder, while neoplastic factors, congenital abnormalities, and underlying diseases also participate in its pathological process. The following discussion is divided into two aspects: classification of pathological causes and core mechanisms: (1) Stone-related factors: Deposition of cholesterol stones is a common metabolic factor leading to gallbladder enlargement. Stones in the gallbladder may be chronically impacted in the cystic duct or neck (4), obstructing bile excretion, increasing intragallbladder pressure, and causing progressive dilation. Multiple stones may move and intermittently block the cystic duct (10); their mechanical stimulation and abnormal bile components can induce acute and chronic inflammatory reactions in the gallbladder wall, promoting progressive gallbladder enlargement and the formation of gallbladder hydrops (6) or rare extra-gallbladder cysts (13). (2) Inflammatory lesions: The pathological manifestations of acute cholecystitis include congestion and edema of the gallbladder wall with neutrophil infiltration (13); in some cases, aggregates of siderophages and foreign-body-type multinucleated giant cells can be observed (2). Chronic cholecystitis is characterized by chronic inflammatory cell infiltration and fibrous tissue proliferation (7). Among them, xanthogranulomatous cholecystitis is associated with the formation of granulomas in the gallbladder, which can further develop into complications such as gallbladder perforation and liver abscess. Chronic gangrenous cholecystitis (9) also contributes to the formation of giant gallbladder through gallbladder wall fibrosis and functional impairment. (3) Neoplastic factors: Progressive growth of malignant tumors such as well-differentiated gallbladder adenocarcinoma (9) and biliary intraductal tubulovillous neoplasms of the gallbladder (12) can lead to gradual enlargement of the gallbladder over time, and can also cause gallbladder dilation through space-occupying effects or impairment of gallbladder emptying mechanisms. Although neoplastic factors for giant gallbladder are relatively rare, the possibility of malignant lesions should be vigilant. (4) Other factors: Congenital gallbladder enlargement may be accompanied by intramural chronic inflammation, resulting in abnormal gallbladder morphology. It has been found that in such patients, serum levels of VEGF and VEGF mRNA gene expression are increased, suggesting that the pathological progression is related to neovascularization (8). In addition, liver diseases such as progressive familial intrahepatic cholestasis (14) are also associated with gallbladder enlargement without any obstructive causes. In another report, localized non-malignant adenomyomatosis was found in the gallbladder neck, accompanied by gallbladder neck ganglion cell depletion, which further led to gallbladder dilation (7); this report also mentioned that it has a certain relationship with diabetes mellitus. The patient in this case had a history of cryptogenic cirrhosis, which may have played a dual role in the development of the giant gallbladder. As a risk factor for cholelithiasis (15), cirrhosis can promote stone formation and biliary stasis through altered bile composition and gallbladder dysmotility (16). Concurrently, the fibrotic remodeling of the gallbladder wall induced by cirrhosis likely reduced its elasticity, rendering the organ more susceptible to persistent dilatation in the setting of obstruction. Thus, cirrhosis may have served not only as a metabolic basis for stone formation but also as a contributing factor to the morphologic evolution of the giant gallbladder.In summary, it can be hypothesized that the pathological essence of giant gallbladder is a vicious cycle of “gallstone-inflammation-structural changes of the gallbladder wall”. Gallstone obstruction causes cholestasis, which stimulates acute and chronic inflammation of the gallbladder wall, thereby leading to structural abnormalities such as fibrous tissue proliferation, cholesterol deposition, and intestinal metaplasia; in turn, gallbladder wall fibrosis can further impede bile excretion, resulting in persistent dilation. In addition, tumor occupation, congenital developmental abnormalities, complications (such as perforation), and comorbidity (such as cirrhosis) can further exacerbate abnormal gallbladder morphology and function by impairing the normal physiological function of the gallbladder or aggravating local inflammation.

Currently, the surgical treatment evaluation for patients with acute cholecystitis mainly relies on scales such as the Parkland Gallbladder Score (PGS), the Tokyo Guidelines 2018 for acute cholecystitis, and the American Association for the Surgery of Trauma—Emergency General Surgery (AAST EGS) acute cholecystitis score. However, none of these guidelines incorporate the impact of gallbladder size on treatment decisions, and the treatment protocols for giant gallbladder (GGB) still require further standardization. In eligible patients, cholecystectomy constitutes the definitive therapy for GGB (7), with the objectives of eliminating infection, resolving obstruction, and lowering the long-term malignancy risk (16). The choice of surgical approach should be individualized based on a comprehensive evaluation of multiple factors: For patients with GGB where the short diameter or thickness of the gallbladder is within an acceptable range, with mild inflammation and no severe adhesions, laparoscopic cholecystectomy (LC) is the preferred approach. Single-stage laparoscopic gallbladder decompression and cholecystectomy is a feasible option for patients presenting with significant gallbladder tension, provided there is minimal local inflammation and only loose adhesions (17). When faced with large gallstones, gallbladder edema, or wall thickening that precludes easy retrieval, the surgeon may opt to deliver the gallbladder and stones intact through an extended umbilical incision (2, 18). Furthermore, for surgeons with extensive experience, giant gallstones do not constitute an absolute indication for open cholecystectomy, and LC should remain the preferred option for GGB patients (16). Given that laparoscopic resection may be difficult for giant gallbladders with severe adhesions, a significantly increased transverse diameter, or marked wall thickening—sometimes necessitating conversion to open surgery (4)—direct open cholecystectomy is therefore a rational consideration for such patients (10, 12). This is because a excessively large gallbladder limits the operative space in laparoscopy and may be accompanied by severe adhesions (13), necrosis, or malignancy (9, 12); in such cases, open surgery ensures a clear operative field and procedural safety. In patients unsuitable for immediate surgery due to severe localized inflammation and compromised general condition, a staged approach is advised: initial percutaneous transhepatic gallbladder drainage (PTGBD) under imaging guidance to control sepsis, decompress the gallbladder, and optimize the patient's condition (13). Definitive surgery (laparoscopic or open cholecystectomy) can then be scheduled electively, offering a safer and more effective management pathway.For inoperable giant gallbladder patients (e.g., with severe cirrhosis and limited survival), the treatment paradigm must shift to palliation. Endoscopic internal drainage via duodenocholecystostomy or gastrocholecystostomy is a viable option to achieve definitive symptom control without the long-term burdens of external drainage. The decision for this approach requires expert endoscopic judgment, balancing its benefit of permanent internal drainage against procedural risks such as leakage and bleeding.In summary, the selection of surgical approach for giant gallbladder is influenced by multifaceted factors: (1) Gallbladder-related factors: stone characteristics (size and number), gallbladder wall edema, the degree of peripheral adhesions, and complications (e.g., gallbladder gangrene, perforation, or tumor). (2) Patient-related factors: age, cardiopulmonary status, comorbidities (such as cirrhosis and diabetes), overall condition, and surgical tolerance. Neoplastic lesions, regardless of size, require radical open surgery; if combined with bile duct or liver involvement, additional procedures may be needed (4). Additionally, the surgeon's experience is critical in determining the approach. The case reported here illustrates how surgical expertise guided the decision to opt for a safer, two-stage strategy: the patient first underwent percutaneous gallbladder drainage to drain a large amount of purulent bile and relieve the obstruction, and then underwent laparoscopic cholecystectomy after her systemic condition improved. The patient recovered well postoperatively.

## Conclusion

This article reports a case of giant gallbladder in a 69-year-old female, whose main manifestations were nausea and vomiting caused by pyloric obstruction due to compression by a giant gallbladder secondary to cholelithiasis and chronic cholecystitis. Combined with 13 cases reviewed in the literature, it was found that giant gallbladder is relatively more prevalent in females and middle-aged and elderly populations, with diverse clinical symptoms; its pathological causes are centered on cholelithiasis and acute/chronic inflammation. In this particular case, cirrhosis may have served as one of the pathological factors contributing to the giant gallbladder. Current guidelines have not yet incorporated the impact of gallbladder size on treatment. Treatment is mainly based on surgical resection, and the choice of surgical approach should be comprehensively determined according to gallbladder size, stone characteristics, degree of inflammation, and comorbidities (e.g., severe acute cases or tumors often require open surgery; mild chronic cases may attempt laparoscopy; some cases may undergo initial drainage followed by two-stage surgery). Herein, we report the clinical diagnosis and treatment process of a case of giant gallbladder and review its clinical manifestations, pathogenesis, and pathological findings, aiming to provide clinical insights and experience for other physicians to deepen their understanding of this disease.

## Data Availability

The original contributions presented in the study are included in the article/Supplementary Material, further inquiries can be directed to the corresponding author.
